# Evodiamine Inhibits *Helicobacter pylori* Growth and *Helicobacter pylori*-Induced Inflammation

**DOI:** 10.3390/ijms22073385

**Published:** 2021-03-25

**Authors:** Ji Yeong Yang, Jong-Bae Kim, Pyeongjae Lee, Sa-Hyun Kim

**Affiliations:** 1 Division of Crop Foundation, National Institute of Crop Science (NICS), Rural Development Administration (RDA), Wanju 55365, Korea; yjy90@korea.kr; 2 Department of Biomedical Laboratory Science, College of Health Sciences, Yonsei University, Wonju 26493, Korea; kimjb70@yonsei.ac.kr; 3 School of Oriental Medicine and Bio Convergence Sciences, Semyung University, Jaecheon 27136, Korea; pjlee1@semyung.ac.kr; 4 Department of Clinical Laboratory Science, Semyung University, Jecheon 27136, Korea

**Keywords:** *Evodia rutaecarpa*, evodiamine, *Helicobacter pylori*, IL-8, inflammation, natural compound, NF-κB

## Abstract

*Helicobacter pylori* (*H. pylori*) classified as a class I carcinogen by the World Health Organization (WHO) plays an important role in the progression of chronic gastritis and the development of gastric cancer. A major bioactive component of *Evodia rutaecarpa*, evodiamine, has been known for its anti-bacterial effect and anti-cancer effects. However, the inhibitory effect of evodiamine against *H. pylori* is not yet known and the inhibitory mechanisms of evodiamine against gastric cancer cells are yet to be elucidated concretely. In this study, therefore, anti-bacterial effect of evodiamine on *H. pylori* growth and its inhibitory mechanisms as well as anti-inflammatory effects and its mechanisms of evodiamine on *H. pylori*-induced inflammation were investigated in vitr. Results of this study showed the growth of the *H. pylori* reference strains and clinical isolates were inhibited by evodiamine. It was considered one of the inhibitory mechanisms that evodiamine downregulated both gene expressions of replication and transcription machineries of *H. pylori*. Treatment of evodiamine also induced downregulation of urease and diminished translocation of cytotoxin-associated antigen A (CagA) and vacuolating cytotoxin A (VacA) proteins into gastric adenocarcinoma (AGS) cells. This may be resulted from the reduction of CagA and VacA expressions as well as the type IV secretion system (T4SS) components and secretion system subunit protein A (SecA) protein which are involved in translocation of CagA and VacA into host cells, respectively. In particular, evodiamine inhibited the activation of signaling proteins such as the nuclear factor κ-light-chain-enhancer of activated B cells (NF-κB) and the mitogen-activated protein kinase (MAPK) pathway induced by *H. pylori* infection. It consequently might contribute to reduction of interleukin (IL)-8 production in AGS cells. Collectively, these results suggest anti-bacterial and anti-inflammatory effects of evodiamine against *H. pylori*.

## 1. Introduction

*Helicobacter pylori* (*H. pylori*) infection has been known to be a common cause among certain factors responsible for high rates of gastric cancer including age, family history, smoking, etc. [1,2]. Therefore, a concerted effort for eradication of *H. pylori* infection still seems to be necessary for health promotion worldwide.

DNA replication and transcription are vital steps for survival and propagation of all known living organisms including *H. pylori*. DnaA, a chromosomal replication initiator protein, forms the complex with DnaB, DNA-unwinding helicase. DnaG primase synthesizes primers [3,4]. DNA polymerase III consists of core polymerases (DnaE, DnaQ and HolE), sliding clamp (DnaN) and clamp-loader (DnaX, HolA, HolB, HolC and HolD) [5]. Bacterial RNA polymerase consists of five subunits [6]. The gene *rpo*A and *rpo*B encode the α and β subunit, respectively. Both *rpo*N and *rpo*D gene encode the σ factors.

One of the essential factors for successful colonization of *H. pylori* in the strongly acidic gastric lumen is the enzyme urease, which is a heterodimer composed of structural subunits UreA and UreB [7,8,9,10]. Once *H. pylori* survives in acidic pH of lumen during the early stages of gastric infection, the bacterium reaches the safe mucus layer and starts colonizing the gastric mucosa [11].

*H. pylori*, which has successfully colonized in gastric epithelial cells, induces an immune response through mechanisms such as the secretion of various inflammatory cytokines [12]. Especially, IL-8 is one of the major inflammatory chemokines. *H. pylori*-mediated IL-8 secretion in gastric epithelial cells requires activation of the nuclear factor κ-light-chain-enhancer of activated B cells (NF-κB) [13,14]. Representative virulence factors contributing to these immune responses as well as pathogenesis associated with *H. pylori*-infections are cytotoxin-associated gene A (CagA) and vacuolating cytotoxin A (VacA).

CagA is injected into host cells by the type IV secretion system (T4SS) comprising 12 subunits including 11 essential proteins (VirB1~VirB11) and VirD4 [15,16,17]. The injected CagA into host cell leads full activation of the mitogen-activated protein kinase (MAPK) pathway [18,19], which may act as the upstream signaling for the activation of NF-κB [20]. Secretion system subunit protein A (SecA) is an ATPase that provides energy for translocation into the cell membrane of VacA [21,22,23,24]. The VacA protein triggers intracellular signal transductions related to inflammation, vaculoation and apoptosis.

*Evodia rutaecarpa* is commonly used as herbal remedy in traditional Chinese medicine for the treatment of abdominal pain, dysentery or amenorrhea. We have previously described the minimum inhibitory concentrations (MICs) of the different methanol concentration extracts against *H. pylori* and the effect on virulence factors of *H. pylori* [25]. Major bioactive ingredients of *Evodia rutaecarpa* include evocarpine, evodiamine, etc. [26].

**Figure 1:**
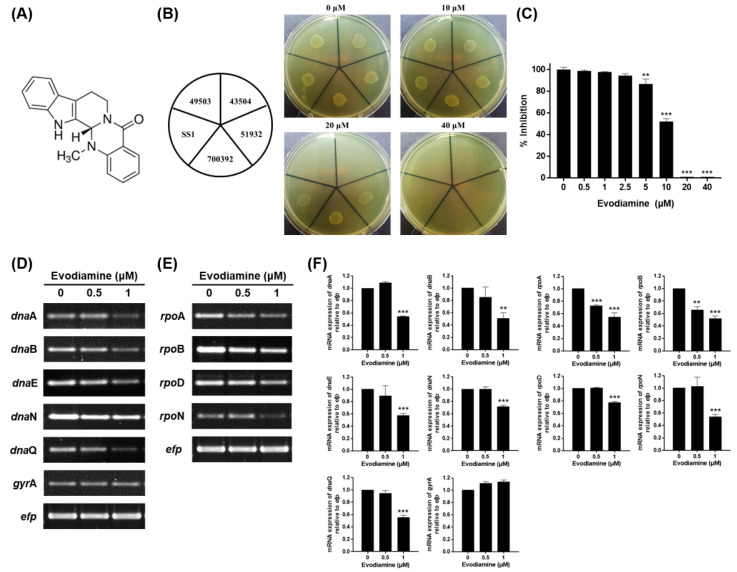
Anti-bacterial activity of evodiamine against *H. pylori* and downregulation of replication and transcription genes of *H. pylori*. (**A**) Chemical structure of evodiamine. (**B**) The MIC of evodiamine against five *H. pylori* reference strains (ATCC 49503, ATCC 43504, ATCC 51932, ATCC 700392 and SS1) was determined by agar dilution method. (**C**) The MIC of evodiamine against *H. pylori* ATCC 49503 strain was confirmed by broth dilution method. Optical density of bacterial broth was measured at 600 nm wavelength by spectrophotometry. *H. pylori* was treated with indicated concentrations of evodiamine (0.5 and 1 μM) for 72 h. RNA was subjected to RT-PCR to detect the mRNA expression levels of (**D**) replication machineries (*dna*A, *dna*B, *dna*E, *dna*N, *dna*Q and *gyr*A) and (**E**) transcription machineries (*rpo*A, *rpo*B, *rpo*D and *rpo*N). The expression of *efp* was used as an internal control. (**F**) Each band intensity was normalized to *efp*. Data are presented as mean ± SEM of three independent experiments and analyzed by Student’s *t*-test (** *p* < 0.01 and *** *p* < 0.001).

Evodiamine has been reported to exhibit numerous pharmacological effects including vasodilatory [27], anti-obesity [28], anti-allergic [29], anti-inflammatory [30] and anti-cancer effects [31,32,33] and chemical structure of evodiamine is shown in Figure 1A. In this study, effects of evodiamine on the expressions of the MAPK and NF-κB-mediated signaling pathway induced by virulence factors of *H. pylori*, as well as its inhibitory effect of evodiamine on *H. pylori* growth and its inhibitory mechanisms were investigated in vitro.

## 2. Results

### 2.1. Inhibitory Effect of Evodiamine on the Growth of H. pylori by Downregulation of Replication and Transcription Genes

An agar dilution test was performed to determine the minimum inhibitory concentration (MIC) of evodiamine against clinical isolates collected from gastric biopsies as well as five *H. pylori* reference strains (ATCC 49503, ATCC 43504, ATCC 51932, ATCC 700392 and SS1). As a result, the MIC of evodiamine against ATCC 49503 and 43504 strains was 20 μM and that of ATCC 51932, 700392 and SS1 was 40 μM (Figure 1B). Among the 50 clinical isolates, the MIC of 42% (21/50) was 20 μM, 20% (10/50) was 10 μM, 14% (7/50) was less than 5 μM, 14% (7/50) was 40 μM and 10% (5/50) was more than 80 μM (Table 1). Because the bacteria were grown in the broth for subsequent experiments, the broth dilution test was conducted to determine the MIC of evodiamine against *H. pylori* ATCC 49503, representatively. Bacterial growth suppression was observed at 5 μM and significant inhibition was observed above 10 μM (Figure 1C). Based on these results, it was demonstrated that evodiamine has an anti-bacterial effect on *H. pylori*. In particular, these results showed the anti-bacterial effect of evodiamine was similar on *H. pylori* clinical isolates as well as the reference strains.

To elucidate how evodiamine inhibits the growth of *H. pylori*, expressions of replication and transcription machinery of *H. pylori* were evaluated. The results show that the mRNA expressions of *dna*A, *dna*B, *dna*E, *dna*N and *dna*Q among the replication machinery of *H. pylori* were decreased in *H. pylori* treated with sub-MIC levels of evodiamine, although the expression of the *gyr*A gene remained constant compared to the evodiamine-non-treated *H. pylori* (Figure 1D,F). Furthermore, evodiamine suppressed the *rpo*A, *rpo*B, *rpo*D and *rpo*N expressions associated with transcription machinery of *H. pylori* compared to the evodiamine-non-treated *H. pylori* (Figure 1E,F). Therefore, the reason why the evodiamine inhibits the growth of *H. pylori* is in part that the replication and transcription factors necessary for the growth of bacteria were suppressed by evodiamine treatment.

### 2.2. Downregulation of Urease in H. pylori Treated with Evodiamine

**Figure 2:**
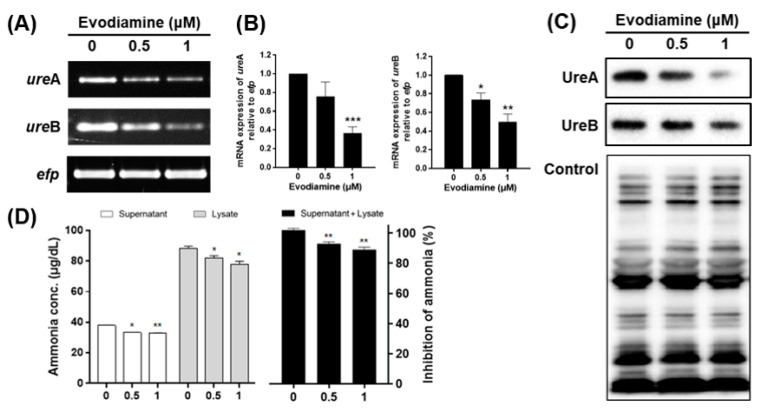
The inhibitory effects of evodiamine on the expression and activity of urease in *H. pylori*. (**A**) *H. pylori* was treated with indicated concentrations of evodiamine (0.5 and 1 μM) for 72 h. RNA was subjected to RT-PCR to detect the mRNA expression levels of urease subunits (*ure*A and *ure*B). The expression of *efp* was used as an internal control. (**B**) Each band intensity was normalized using to *efp*. (**C**) The cell lysates were subjected to Western blot to detect UreA and UreB. The rabbit anti-*H. pylori* polyclonal antibody was used as an internal control. (**D**) After 72 h, the supernatant and cell lysate were collected and then urease activity was determined by measuring ammonia levels. Each band intensity was normalized by protein quantification. Data were presented as mean ± SEM of three independent experiments and analyzed by Student’s *t*-test (* *p* < 0.05, ** *p* < 0.01 and *** *p* < 0.001).

The expression of urease is essential for *H. pylori* to colonize the gastric mucosa to survive in the highly acidic environment. Therefore, it was examined whether evodiamine affects the urease of *H. pylori* which in turn affects bacterial attachment of gastric mucosa. The mRNA and protein expressions of the α and β subunit consisting the urease of *H. pylori* were confirmed by RT-PCR and Western blot, respectively. As a result, it was confirmed that the mRNA expressions of both *ure*A and *ure*B significantly decreased with the treatment of evodiamine (Figure 2A,B). The expression of UreA protein was decreased by evodiamine in a dose-dependent manner and that of UreB seemed to be slightly decreased (Figure 2C).

To confirm the effect of the reduction of UreA and UreB expressions on urease activity, the amount of ammonia produced by urease activity was measured. The amount of ammonia in the media supernatant was 38.1 μg/dL at 0 μM, 33.4 μg/dL at 0.5 μM and 32.8 μg/dL at 1 μM. The amount of ammonia in cell lysate was 88.5, 82.0 and 78.0 μg/dL at 0, 0.5 and 1 μM, respectively. Total amount of ammonia decreased by 7% at 0.5 μM and 11% at 1 μM treated group when compared to the untreated group (Figure 2D). The production of ammonia in the supernatant and cell lysate seems to be a result of the urease activity of *H. pylori*, which is thought to be caused by inhibition of UreA and UreB expressions by evodiamine. In summary, these data indicate that evodiamine may restrict colonization and survival of *H. pylori* in the gastric mucosa by reducing the expression of urease.

### 2.3. Reduced CagA and VacA Translocation to AGS Cells by Evodiamine

**Figure 3:**
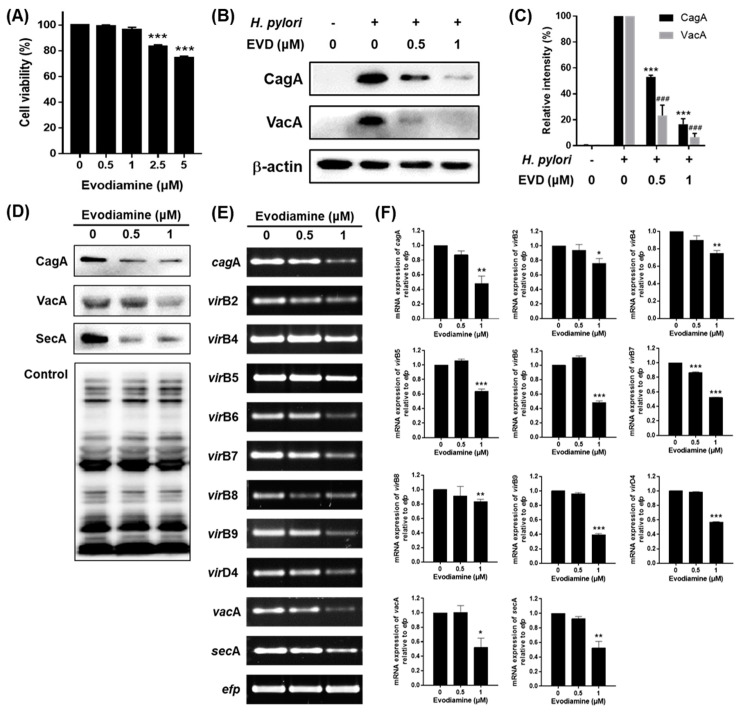
The inhibitory effects of evodiamine on CagA and VacA translocation to the gastric cell line. (**A**) AGS gastric cancer cells were treated with indicated dose of evodiamine (0.5, 1, 2.5 and 5 μM) for 24 h and cell viability was measured by the WST assay. AGS cells were infected with *H. pylori* (200 MOI) and treated with indicated concentrations of evodiamine (0.5 and 1 μM) for 24 h. (**B**) After incubation, the cell lysates were collected and Western blot was then performed to detect CagA and VacA proteins. β-actin was used as an internal control. (**C**) Each band intensity was normalized to β-actin. *H. pylori* was treated with indicated concentrations of evodiamine (0.5 and 1 μM) for 72 h. (**D**) The cell lysates were subjected to Western blot to detect CagA, VacA and SecA. The rabbit anti-*H. pylori* polyclonal antibody was used as an internal control. (**E**) RNA was subjected to RT-PCR to detect the mRNA expression level of *cag*A, T4SS components, *vac*A and *sec*A. The expression of *efp* was used as an internal control. (**F**) Each band intensity was normalized to *efp*. Data were presented as mean ± SEM of three independent experiments and analyzed by Student’s *t*-test (^###^ *p* < 0.001, * *p* < 0.05, ** *p* < 0.01 and *** *p* < 0.001).

When *H. pylori* adheres to the gastrointestinal mucosa, it secretes a number of virulence factors that can destroy gastric epithelial cells causing gastrointestinal disease. We examined whether evodiamine affects the amount of CagA and VacA proteins translocated into host cells. AGS cells were exposed to live bacteria at a multiplicity of infection (MOI) of 200 for 24 h in presence of evodiamine at two concentrations that do not affect cell viability (Figure 3A), then CagA and VacA proteins were detected in the lysates of *H. pylori*-infected AGS cells. However, both proteins were significantly decreased by evodiamine treatment (Figure 3B,C). Especially, VacA protein was dramatically decreased even by low dose of evodiamine treatment.

To find out why the amount of translocated proteins of CagA and VacA decreased, the mRNA and protein expression levels of CagA, VacA and each secretion system in *H. pylori* treated with evodiamine were assessed by RT-PCR and Western blot. Both the mRNA and protein levels of CagA and VacA in *H. pylori* were decreased by evodiamine treatment (Figure 3D–F). Furthermore, the mRNA expressions of *vir*B2, *vir*B4, *vir*B5, *vir*B6, *vir*B7, *vir*B8, *vir*B9 and *vir*D4 were decreased by evodiamine treatment (Figure 3E,F). The mRNA and protein expressions of SecA in *H. pylori* were also suppressed by evodiamine treatment (Figure 3D–F).

Collectively, these data indicated that evodiamine not only reduced the expressions of CagA and VacA in *H. pylori*, but also reduced the translocation of CagA and VacA into host cells by downregulation of both the T4SS components required for CagA injection and SecA necessary for VacA secretion.

### 2.4. Decrease of Inflammatory Cytokines Induced by H. pylori Infection via Inhibition of MAPK and NF-κB Activation by Evodiamine

**Figure 4:**
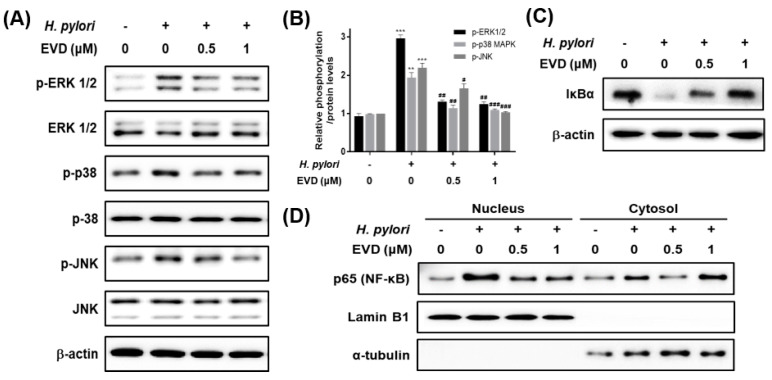
The inhibitory effects of evodiamine on *H. pylori*-induced activation of MAPK and NF-κB and degradation of IκBα in AGS cells infected with *H. pylori*. AGS cells were infected with *H. pylori* (200 MOI) and treated with indicated concentrations of evodiamine (0.5 and 1 μM) for 12 h. (**A**) After incubation, cell lysates were collected and Western blot analysis of phosphorylated and total ERK 1/2, JNK and p38 was performed. β-actin was used as an internal control. (**B**) Each band intensity was normalized to β-actin. Data were presented as mean ± SEM. ** *p* < 0.01 and *** *p* < 0.001 vs. uninfected control. ^#^ *p* < 0.05, ^##^ *p* < 0.01 and ^###^ *p* < 0.001 vs. *H. pylori*-infected control without evodiamine treatment. (**C**) Western blot analysis of IκBα was performed. β-actin was used as an internal control. (**D**) Cell lysates were separated into nuclear and cytosolic fractions, then performed to Western blot for NF-κB. Lamin B1 was used as an internal control for nuclear fraction and α-tubulin was used as an internal control for cytosolic fraction. EVD, evodiamine.

To investigate whether evodiamine inhibits *H. pylori*-induced activation of NF-κB through the MAPK signaling pathways and inflammatory responses, AGS cells infected with *H. pylori* were treated with evodiamine for 12 h (MAPK, IκB and NF-κB) and 24 h (IL-8). As shown in Figure 4A,B, total ERK1/2, p38 and JNK were not changed by *H. pylori* and/or evodiamine treatment in AGS cells. However, phospho-forms of ERK1/2, p38 and JNK increased more than 2-fold by *H. pylori* infection in AGS cells. Evodiamine, on the other hand, inhibited phosphorylation of ERK1/2, p38 and JNK induced by *H. pylori* from the concentration of 0.5 μM of evodiamine.

To determine whether NF-κB activated by *H. pylori* is inhibited by treatment with evodiamine, IκBα degradation and NF-κB activation were confirmed by Western blot. IκBα protein was decreased by *H. pylori* infection and recovered the IκBα protein level by evodiamine (Figure 4C). It indirectly indicated that evodiamine reduced NF-κB activation by *H. pylori* infection. In addition, Western blot results for NF-κB in each fraction showed that NF-κB in the nucleus was increased by *H. pylori* infection, but it was reduced by evodiamine treatment (Figure 4D).

**Figure 5:**
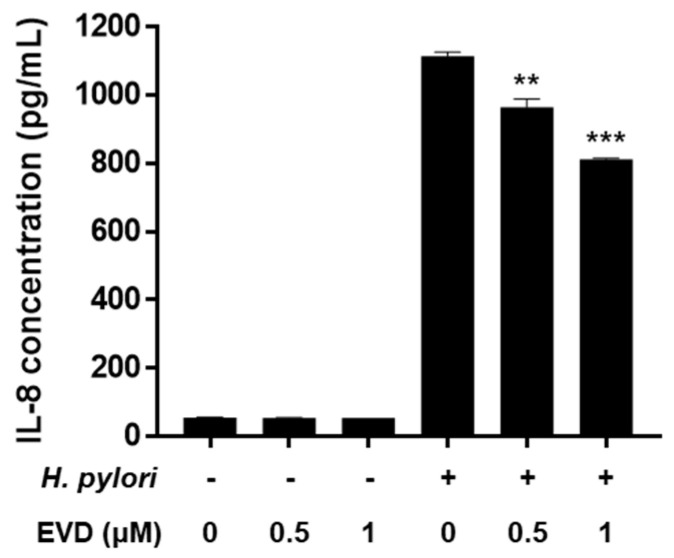
Inhibitory effect of evodiamine on IL-8 secretion induced by *H. pylori* in AGS cells. AGS cells were uninfected or infected with *H. pylori* (200 MOI) with indicated concentrations of evodiamine (0.5 and 1 μM). Culture medium were collected after 24 h and ELISA was performed to determine the IL-8 concentration. Data were presented as mean ± SEM of three independent experiments and analyzed by Student’s *t*-test (** *p* < 0.01 and *** *p* < 0.001). EVD, evodiamine.

NF-κB has a dominant role in *H. pylori*-induced IL-8 secretion from gastric epithelial cells. The protein level of IL-8 was unaffected by evodiamine treatment alone. However, *H. pylori*-infection increased IL-8 level and evodiamine significantly decreased IL-8 production by 27% compared to the *H. pylori*-infected AGS cells without evodiamine (Figure 5). Collectively, these results suggested that evodiamine inhibited upregulated MAPK signaling pathway and NF-κB by *H. pylori* infection, which may lead to reduction of IL-8 inflammatory cytokine in the gastric epithelial cells.

## 3. Discussion

*H. pylori* infection is one of the critical risk factors for gastric cancer [34] and is responsible for 75% of all the gastric cancer cases [35,36]. However, numerous reports have suggested the prevalence of clarithromycin resistance and the limitation of current first-line therapy [37,38,39]. Therefore, development of a new therapeutic or supportive agent to help eradication of *H. pylori* is necessary. Evodiamine has anti-bacterial effect on *Klebsiella pneumoniae*, a Gram-negative bacterium [40]. In addition, evodiamine was suggested to have anti-inflammatory effect on macrophage and different types of epithelial cells through inhibition of NF-κB signaling [30,41,42]. In this study, thus, the inhibitory effect of evodiamine on *H. pylori* growth and *H. pylori*-induced inflammation was investigated.

The MIC of evodiamine against *H. pylori* reference strains was 20 μM (6.07 μg/mL) or 40 μM (12.13 μg/mL) in the agar dilution method (Figure 1B), whereas that was 5 μM (1.52 μg/mL) in the broth dilution method (Figure 1C). Drugs can diffuse only on the surface of agar plate; however, in broth medium, they can act continuously and directly on bacteria as they grow. It is estimated that the reason why the MIC in the broth test showed lower than that of agar test. *Evodiae rutaecarpa* extract has been known that it contains quinolone alkaloids such as evocarpine and dihydroevocarpine and indoloquinazoline alkaloids such as evodiamine and rutaecarpine. Norio Hamasaki et al. reported that the mixture fractionated from *Evodiae rutaecarpa* containing evocarpine and 1-methyl-2-[(Z)-7-trideceny1]10-4-(1*H*)-quinolone (10:1), the quinolone alkaloids compounds, inhibited the growth of *H. pylori* at the lowest concentration, 2 μg/mL, among the fractions by the disk method [43]. Along with other substances constituting the crude extract of *Evodiae rutaecarpa*, evodiamine also seems to affect the anti-*H. pylori* activity of the extract.

From *H. pylori* isolated from patients, it was confirmed that bacterial growth was inhibited at even lower concentrations than that of reference strains as well as about half of them showed the same MIC as the reference strains (Table 1). The antibiotic susceptibility results according to the Clinical & Laboratory Standard Institute (CLSI) guidelines for 50 clinical strains were shown in Supplementary Data 1. Among the isolates, 7 of 8 clarithromycin-resistant strains and 10 of 11 strains which are resistant to more than one antibiotic were inhibited by evodiamine at a concentration of less than 40 μM (data not shown). The MIC in the broth dilution method was lower than the result from the agar method (Figure 1B,C). It suggests that low concentrations of evodiamine may have anti-bacterial effect on *H. pylori* in clinical use of evodiamine. Furthermore, *H. pylori* which could not be eliminated due to antibiotic resistance, is also thought to be effectively eradicated by evodiamine.

Evodiamine inhibited *H. pylori* growth via downregulation of replication and transcription machinery of *H. pylori* essential for survival (Figure 1D–F). DnaN and RpoB, especially, have been used for target of antibiotics. *Streptomyces*-derived griselimycin is highly active against *Mycobacterium tuberculosis* by inhibiting the DNA polymerase sliding clamp DnaN [44]. In addition, rifampin targets RpoB to inhibit transcription of *M. tuberculosis* [45]. It has been reported that increased expression of RpoB is associated with survival and growth in rifampin [46]. Collectively, as it is indispensable for replication or transcription of bacteria, this result suggests that evodiamine may be one of the mechanisms that inhibit the growth of *H. pylori*. Furthermore, reduction of transcription means that proteins produced by *H. pylori* including virulence factors may also be reduced, which is consistent with this study.

Downregulation of urease subunits by evodiamine may contribute to inhibit initial colonization of *H. pylori* to the gastric epithelium and survive under acidic conditions (Figure 2). It has been reported that the methanol extract of *Evodiae rutaecarpa* inhibits the urease activity of *H. pylori* [25]; however, it has been not known which substance affects yet. Rho TC et al. demonstrated that quinolone alkaloids had no inhibitory effect on urease activity of *H. pylori* [47]. Based on Figure 2D, it is assumed that evodiamine affects the inhibition of urease activity. Especially, decrease of UreB expression by evodiamine results in the reduced enzymatic activity of urease in *H. pylori*. Moreover, urease activity may be responsible for damage to the gastric epithelium through interaction with the immune system. Fan X et al. reported that urease binds to major histocompatibility complex (MHC) class II and induces apoptosis of gastric epithelial cells [48]. Ellen J et al. also reported that the UreB interacts CD74 and that induces NF-κB activation and IL-8 production [49]. It implies that urease reduced by evodiamine potentially leads to the weakened immune responses during infection.

Evodiamine suppressed translocation of CagA and VacA into host cells, which may result from downregulation of the expressions of CagA and VacA as well as the secretion systems which are involved in translocation of them (Figure 3). The T4SS in *H. pylori* contributes to gastric cancer development by mediating entry of CagA [50]. In this study, the mRNA expressions of *vir*B2, *vir*B4-9 and *vir*D4 were decreased by evodiamine treatment (Figure 3E,F). Among components of inner membrane complex (VirB4, VirB6, VirB8 and VirD4), VirB4 and VirD4 are especially required to regulate substrate recruitment and transport it into T4SS [51]. VirB7 and VirB9 are necessary for injection of CagA because they form a trans-membrane pore complex channel to pass the substrate through the periplasmic space and are anchored in the outer membrane [51,52]. T4SS external pilus composed of VirB2 and VirB5 proteins is also essential for making the connection between the recipient and donor cells [51,52,53]. Translocation of VacA across the inner membrane is mediated by Sec-dependent T5_a_SS [21]. Among Sec-dependent proteins, SecA is an ATPase which is bound to the complex of inner membrane bound Sec-related family proteins [54]. In the current study, the mRNA and protein levels of SecA were downregulated by evodiamine (Figure 3D–F). Taken together, since evodiamine not only reduced the production of *H. pylori*-secreting CagA and VacA proteins, but also reduced the components of T4SS and T5_a_SS, it is thought that the amount of CagA and VacA proteins that have translocated into the host cell may be reduced by evodiamine.

Chronic *H. pylori* infection leads to inflammation of the gastric mucosa. The chemokine, IL-8, whose secretion is induced by *H. pylori* plays a central role in the pathogenesis of gastritis. In the present study, evodiamine inhibited the activation of MAPK and the nuclear translocation of NF-κB in *H. pylori*-infected AGS cells (Figure 4). In addition, the protein level of IL-8 was significantly suppressed by evodiamine treatment (Figure 5), in parallel with the degradation of IκBα and inhibition of NF-κB activation as shown as Figure 4. CagA of *H. pylori* contributes to the secretion of IL-8 through MAPK and NF-κB activation [18]. IL-8, a potent leukocyte chemoattractant and activating agent [55], induces the release of reactive oxygen species (ROS) [7,56]. ROS production in gastric epithelial cells is enhanced by CagA proteins of *H. pylori* [57] and the oxidative stress stimulates MAPK such as ERK, JNK and p38 and up-regulates transcription of NF-κB [56,58].

Furthermore, it has been also reported that VacA stimulation induces NF-κB activation in human eosinophils which can secrete IL-8 in an NF-κB-dependent manner [59]. However, Junzo Hisatsune et al. reported that VacA-induced IL-8 release was not observed in AGS cell lines [60]. Collectively, reduction of CagA translocation into host cells by evodiamine as shown in Figure 3B,C, in turn, leads to downregulation of MAPK activation, inhibition of NF-κB activation and eventually inhibition of IL-8 secretion induced by *H. pylori* infection. In addition, it is expected that evodiamine provides even more potent anti-inflammatory effect on *H. pylori*-induced inflammation according to the result of dramatically decreased translocated VacA protein into AGS cells by evodiamine (Figure 3B,C).

Further studies are required to fully demonstrate anti-inflammatory mechanism of evodiamine against *H. pylori*. The MAPK pathway has been shown to mediate the activator protein-1 (AP-1) activation during *H. pylori* infection in gastric epithelial cells [61]. Therefore, it would be explained the complete anti-inflammatory mechanism of evodiamine by confirming the expression changes of AP-1. It would be also interesting to determine anti-inflammatory effect of evodiamine during *H. pylori* infection in lymphocyte cell lines and primary immune cells. In addition, in vivo studies seem to be necessary to evaluate the success of *H. pylori* eradication, anti-inflammatory effect and toxicity of evodiamine.

## 4. Materials and Methods

### 4.1. Bacterial and Mammalian Cell Culture

The *H. pylori* reference strains of ATCC 49503, ATCC 43504, ATCC 51932 and ATCC 700392 were purchased from the American Type Culture Collection (ATCC, Manassas, VA, USA) and the *H. pylori* SS1 strain was obtained from the Korean Type Culture Collection at Gyeongsang National University (Jinju, Korea). Fifty strains of *H. pylori* clinical isolates were isolated from 50 patients undergoing gastroscopic examination to confirm *H. pylori* infection at Yong-In Severance Hospital in Korea. The experiments were conducted with Institutional Review Board approval at Yonsei University Mirae Campus (IRB No. 1041849-201705-BR-056-01). *H. pylori* were grown on Brucella agar plates (BD Biosciences, Franklin Lakes, NJ, USA) supplemented with 10% bovine serum (BRL Life Technologies, Grand Island, NY, USA) under microaerophilic and 100 percent humidity conditions at 37 °C and inspected after three to five days.

AGS gastric adenocarcinoma cells (ATCC CRL-1739) were purchased from the Korean Cell Line Bank (Seoul, Korea) and cultured in Dulbecco’s modified Eagle’s medium (DMEM, BRL Life Technologies) supplemented with 10% fetal bovine serum (FBS, BRL Life Technologies) and streptomycin-penicillin (100 μg/mL and 100 IU/mL) (BRL Life Technologies). Cells were incubated at 37 °C in a humidified atmosphere with 5% CO_2_. The experiments were conducted under Institutional Biosafety Committee approval at Yonsei University Mirae Campus (IBC No. 201909-P-006-01).

### 4.2. Determination of MIC

For agar dilution test, 10 μL of the bacterial suspension (McFarland 2.0) were placed on the Mueller–Hinton agar (BD Biosciences) supplemented with 10% bovine serum including indicated concentrations of evodiamine (Sigma-Aldrich, St Louis, MO, USA). The bacteria were incubated for 72 h and the minimum inhibitory concentration (MIC) was determined based on the lowest concentration of growth inhibition. For broth dilution test, various concentrations of evodiamine (0.5~40 μM) were treated and the bacteria (McFarland 0.5) were incubated for 72 h. All of the solutions were prepared in such a manner that the final dimethylsulfoxide (DMSO) concentration was the same in all treatments. Final optical density (600 nm) of the bacterial suspension was measured by spectrophotometry.

### 4.3. RNA Extraction and Reverse Transcriptase-Polymerase Chain Reaction (RT-PCR)

Cultured *H. pylori* were washed twice with sterile saline and total RNA was extracted using Trizol reagent (Invitrogen, Carlsbad, CA, USA) as described in the manufacturer’s instructions. The PCR primer sequences used in this study are listed in Table 2 [62,63,64,65,66,67]. *Efp* (elongation factor P) was used as an internal control. The band intensity of PCR product was analyzed with the ImageLab software (Bio-Rad, Hercules, CA, USA).

### 4.4. Protein Extraction and Western Blot

AGS cells were seeded into 90 mm dishes at a density of 2.4 × 10^6^ cells/10 mL in DMEM containing 10% FBS without antibiotics and for 24 h prior to infection. *H. pylori* was added to the cells at a multiplicity of infection (MOI) of 200 in the absence or presence of evodiamine. After 12 h, the infected cells were incubated gentamicin-containing (25 μg/mL) medium before the samples were harvested. The cell or bacteria was lysed with radio immunoprecipitation assay (RIPA) lysis buffer (Millipore, Billerica, MA, USA) containing a protease inhibitor cocktail. The cell lysates were incubated on ice for 10 min, centrifuged at 12,000× *g* at 4 °C for 10 min and then the supernatants were collected.

Antibodies to detect CagA, VacA, Lamin B1, α-tubulin and β-actin were purchased from Santa Cruz Biotechnology (Dallas, TX, USA) and the polyclonal antibody against whole *H. pylori* (ATCC 49503) was produced as previously described [68]. The antibodies to detect PARP, ERK, p-ERK, p38, p-p38, JNK, p-JNK, IκBα and NF-κB were purchased from Cell Signaling Technology (Danvers, MA, USA). The polyclonal antibody against whole *H. pylori* (ATCC 49503) or β-actin was used as an internal control for *H. pylori* or mammalian cell proteins, respectively.

### 4.5. Urease Activity Test

*H. pylori* ATCC 49503 strain was treated with the indicated concentrations of evodiamine (0.5 and 1 μM). After 72 h, 100 μL of medium were collected, centrifuged at 5000× *g* for 10 min and then separated into medium and cells. The cells were lysed with 100 μL of RIPA buffer containing a protease inhibitor at 4 °C for 10 min. The lysates were centrifuged at 12,000× *g* at 4 °C for 10 min and the supernatants were collected. The collected lysates were quantified by Lowry protein assay. The medium was quantified by measuring the optical density at 600 nm using NanoQuant Infinite M200. After adding 5 μL of 20% urea to the specimens respectively, the specimens were incubated at 37 °C for 10 min. Urease activity was determined by using an Asan Set Ammonia kit (Asan Pharmaceutical, Seoul, Korea) to measure ammonia levels according to manufacturer’s instruction. The ammonia concentration of the specimens was calculated using the standard curve.

### 4.6. Subcellular Fractionation

Cellular cytosolic and nuclear fractions were prepared by incubating cells in 400 μL of ice-cold hypotonic solution buffer (20 mM Tris-HCl, 3 mM MgCl_2_ and 10 mM NaCl_2_) with a protease inhibitor cocktail. The lysates were incubated for 10 min on ice and homogenized 40 times with a Dounce homogenizer (Wheaton, Millville, NJ, USA). The cell lysates were centrifuged at 5000× *g* for 10 min at 4 °C and supernatant (cytosolic fraction) was transferred to a new tube and mixed with 5× SDS sample loading buffer. Nuclear pellet was washed once with hypotonic solution buffer to clear the nuclear debris and lysed with 2× SDS sample loading buffer. Before Western blot, nuclear fraction samples were sonicated for 5 s with 20 Hz (Vibracell, Danbury, CT, USA).

### 4.7. Enzyme-Linked Immunosorbent Assay (ELISA)

AGS cells were seeded into 6-well cell culture plates at a density at 2 × 10^5^ cells/2 mL in DMEM containing 10% FBS without antibiotics and for 24 h prior to infection. *H. pylori* was added to the cells at the MOI of 200 in the absence or presence of evodiamine. After 12 h, the infected cells were incubated gentamicin-containing (25 μg/mL) medium before the supernatants were harvested. The supernatant was collected and centrifuged at 5000× *g* at 4 °C for 10 min to remove the host cells and cell debris. To determine the production of IL-8, 100 μL of cell culture supernatants were analyzed by IL-8 human uncoated ELISA kit (Invitrogen) according to manufacturer’s instruction.

### 4.8. Statistical Analysis

Data in the bar graphs are presented as mean ± standard error of mean (SEM). All the statistical analyses were performed using GraphPad Prism 7.0 software (GraphPad Software, San Diego, CA, USA). All the data were analyzed by unpaired Student’s *t*-test and *p* < 0.05 was considered to be statistically significant.

## Figures and Tables

**Table 1:** MIC of evodiamine on *H. pylori* clinical isolates.

Evodiamine Concentration (μM)	Number of Strains (%)	
≤5	7	(14%)
10	10	(20%)
20	21	(42%)
40	7	(14%)
≥80	5	(10%)
Total	50	(100%)

**Table 2:** List of primer sequences and PCR conditions for RT-PCR.

Primers	Sequences (5′-3′)		Product Length (bp)	Annealing Temperature (°C)	Cycles	Reference
	Forward	Reverse				
DnaA	GGGCATGACTTAGCGGTTA	TTAACGAATTGCACGCCAAC	128	55	27	[62]
DnaB	AATGGGCCGTTTATCGTCTC	CAAATCCGCTTGCAACTACG	231	55	27	
DnaE	AATCCACCGGCTCCAAATAC	GCCAAACAAGTGTGGGAGTA	184	55	27	
DnaN	GTTAGCGGTGGTTGAAAACG	CGGTTTCGCTATGCTCAGAA	233	55	27	
DnaQ	CGCATGAAGCTTTGCAAGAA	GCATAGGCTCTATGGCTGAC	244	55	27	
GyrA	GTGCATAGGCGTATTTT	CATTCTGGCTTCAGTGTAACG	246	52	25	
RpoA	AGCGACACGTCTTCAGTAAC	ACAGCACCTTTGATCCCATC	224	55	22	[65]
RpoB	TTTAGGTAAGCGCGTGGATT	AATCAGCTTTGGATGGAACG	301	59	24	
RpoD	TCATCATCATTGCCGACTGG	GTCATGCGCAAACACATTCA	152	55	26	
RpoN	GCCCTTGAAATCGTGCTTAC	ATGATGAGAGCTACCCGACA	250	55	27	
UreA	GCCAATGGTAAATTAGTT	CTCCTTAATTGTTTTTAC	411	40	20	[66]
UreB	TCTATCCCTACCCCACAACC	CCATCCACGAACACATGGTA	252	50	21	
CagA	GTCATAATGGCATAGAACCTGAA	ATTCCCTAGGGCGTCTAAATAA	407	59	21	[63]
VirB2	CAGTCGCCTGACCTCTTTTGA	CGGTCACCAGTCCTGCAAC	156	62	25	
VirB4	GTTATAGGGGCAACCGGAAG	TTGAACGCGTCATTCAAAGC	449	62	37	
VirB5	TACAAGCGTCTGTGAAGCAG	GACCAACCAACAAGTGCTCA	436	62	30	
VirB6	CCTCAACACCGCCTTTGGTA	TAGCCGCTAGCAATCTGGTG	225	62	25	
VirB7	GATTACGCTCATAGGCGATGC	TGGCTGACTTCCTTGCAACA	202	62	25	
VirB8	GTTGATCCTTGCGATCCCTCA	CGCCGCTGTAACGAGTATTG	218	62	25	
VirB9	GCATGTCCTCTAGTCGTTCCA	TATCGTAGATGCGCCTGACC	269	62	25	
VirD4	CCGCAAGTTTCCATAGTGTC	GCGAGTTGGGAAACTGAAGA	263	62	25	
SecA	AAAAATTTGACGCTGTGATCC	CCCCCAAGCTCCTTAATTTC	274	47	27	
VacA	AAACGACAAGAAAGAGATCAGT	CCAGCAAAAGGCCCATCAA	291	57	22	[64]
Efp	GGCAATTTGGATGAGCGAGCTC	CTTCACCTTTTCAAGATACTC	559	59	23	[67]

## Data Availability

The data that support the findings of this study are available from the corresponding author upon reasonable request.

## Supplementary Materials

Supplementary materials can be found at https://www.mdpi.com/1422-0067/22/7/3385/s1.
