# Supplementary material for: Evodiamine Inhibits Helicobacter pylori Growth and Helicobacter pylori-Induced Inflammation
Source: Int J Mol Sci. 2021 Mar 25;22(7):3385. doi: 10.3390/ijms22073385 (PMC8036659; doi:10.3390/ijms22073385)
Supplement: Supplementary file 1 [file ijms-22-03385-s001.pdf]

Supplementary data 1. The antibiotic susceptibility results for the used clinical strains

Clarithromycin	Amoxicillin	Metronidazole	Tetracycline	Levofloxacin
**128**	0.016	**32**	1	0.25
**64**	0.03125	**32**	1	0.125
**64**	0.0625	**16**	2	0.25
**16**	0.25	**32**	0.0625	0.125
**16**	0.0625	2	0.0625	**8**
**16**	0.0625	0.0625	0.03125	**4**
**8**	0.008	2	1	**4**
**2**	0.004	**64**	0.004	**4**
0.25	**1**	**32**	1	0.25
0.004	0.0625	**32**	0.125	**2**
0.5	0.0625	**32**	0.5	**1**
0.004	0.5	**64**	0.004	0.125
0.004	0.004	**32**	0.016	0.0625
0.0625	0.004	**32**	1	0.25
0.03125	0.0625	**32**	0.125	0.25
0.0625	0.0625	**16**	0.016	0.0625
0.03125	0.03125	**16**	0.0625	0.125
0.03125	0.25	**16**	0.0625	0.125
0.016	0.03125	**16**	0.125	0.125
0.004	0.5	**16**	0.03125	0.125
0.004	0.25	**8**	0.0625	0.125
0.004	0.25	**8**	0.03125	0.125
0.25	0.016	2	2	**4**
0.0625	0.5	4	0.0625	**4**
0.0625	0.25	2	0.03125	**4**
0.004	0.25	0.004	0.0625	**4**
0.004	0.03125	0.03125	0.016	**2**
0.125	0.25	2	0.0625	0.125
0.125	0.016	2	2	0.25
0.0625	0.0625	4	0.0625	0.125
0.0625	0.25	2	0.125	0.25
0.03125	0.5	2	0.0625	0.0625
0.03125	0.0625	2	0.125	0.25
0.03125	0.25	2	0.125	0.125
0.03125	0.125	2	0.03125	0.5
0.03125	0.25	4	0.125	0.125
0.03125	0.03125	0.03125	0.125	0.25
0.004	0.004	1	0.004	0.0625
0.004	0.5	0.125	0.0625	0.03125

0.004	0.25	0.0625	0.0625	0.0625
0.004	0.25	2	0.004	0.5
0.004	0.125	0.016	0.016	0.004
0.004	0.125	0.03125	0.125	0.004
0.004	0.0625	1	0.016	0.25
0.004	0.03125	0.004	0.016	0.004
0.004	0.03125	0.008	0.004	0.0625
0.004	0.004	1	0.0625	0.004
0.004	0.004	0.004	0.004	0.004
0.004	0.004	0.5	0.004	0.125
0.004	0.004	0.004	0.004	0.004

Cut-off values of resistance were defined as ≥ 1 μg/mL for clarithromycin, ≥ 1 μg/mL for amoxicillin, ≥ 8 μg/mL for metronidazole, ≥ 4 μg/mL for tetracycline and ≥ 1 μg/mL for levofloxacin.
